# A Wise Resolve

**Published:** 1892-10-01

**Authors:** 


### Passing Topics.

### A WISE RESOLVE.

The authorities of the London Hospital are to be sincerely congratulated upon their resolution to maintain the best traditions of that great institution. The retirement of Mr. Nixon, to whose sterling qualities a tribute has been already paid in the columns of The Hospital, exposed them, without doubt, to the full force of influences as persistently at work in respect of the management of our medical charities, as they are difficult to withstand, and nowhere could we find better testimony to the excellence of the late House Governor's administration than in the resolve that he should have a successor. All we hope is that the "some alterations" in the management which Mr. J. H. Buxton says have been decided upon will tend to consolidate and not to weaken the powers of the Governor's office. It is no disparagement of the gentleman chosen, but the contrary, to say that the task he has been set is one which will call for the exercise of his best abilities. Precisely because Mr. Nixon has been so uniformly and conspicuously successful, his successor must be prepared to be judged by a high standard, and for the same reason it was possible to argue that the qualities the former had displayed betokened a personality it would be well-nigh impossible to replace. Had this conclusion once been admitted, a serious blow would have been struck at the maintenance of that supremacy of lay authority which, as we have recently shown, is of paramount importance to our hospitals as philanthropic institutions, and has been nowhere more prominently illustrated than in the history of the London Hospital.

The fact that the House Committee have held their ground is one that should encourage other lay governing bodies to oppose all attempts to pare away their powers. Committees are not always quite true to themselves or to the interests they serve. The differences which divide the medical notions of a hospital from the lay are real and not imaginary, and it is greatly to be regretted that committeemen are sometimes slow to perceive this fundamental truth. Occasionally they appear far too prone to surrender the position they are charged to defend, in favour, as they are pleased to explain, of the cause of peace. No course could be more mistaken. Whatever the recommendations of individual physicians and surgeons, and we well know how high these can be, the example set by the London Hospital in altogether excluding members of the active medical and surgical staff from the governing committees is essentially a sound one, and it would have been better for medical charity had it been more generally followed. The world of medical philanthropy owes a tribute of thanks to the House Committee of the London Hospital. Its most recent action, whether consciously or unconsciously taken, affords the best protection that could be devised against the encroachments of the professional tide, which recognises no bounds but those imposed by obstacles it cannot overcome, and where these do not exist, threatens something not unlike an inundation. Agricola.

